# Less Is More: Can Low Quantum Capacitance Boost Capacitive Energy Storage?

**DOI:** 10.1021/acs.jpclett.2c02968

**Published:** 2022-11-18

**Authors:** Taras Verkholyak, Andrij Kuzmak, Alexei A. Kornyshev, Svyatoslav Kondrat

**Affiliations:** † Institute for Condensed Matter Physics, National Academy of Sciences of Ukraine, Svientsitskii Street 1, 79011Lviv, Ukraine; ‡ Department for Theoretical Physics, I. Franko National University of Lviv, 79000Lviv, Ukraine; § Department of Chemistry, Molecular Sciences Research Hub, White City Campus, LondonW12 0BZ, United Kingdom; ∥ Thomas Young Centre for Theory and Simulation of Materials, Imperial College London, South Kensington Campus, LondonSW7 2AZ, United Kingdom; ⊥ Institute of Physical Chemistry, Polish Academy of Sciences, 01-224Warsaw, Poland; # Institute for Computational Physics, University of Stuttgart, 70049Stuttgart, Germany

## Abstract

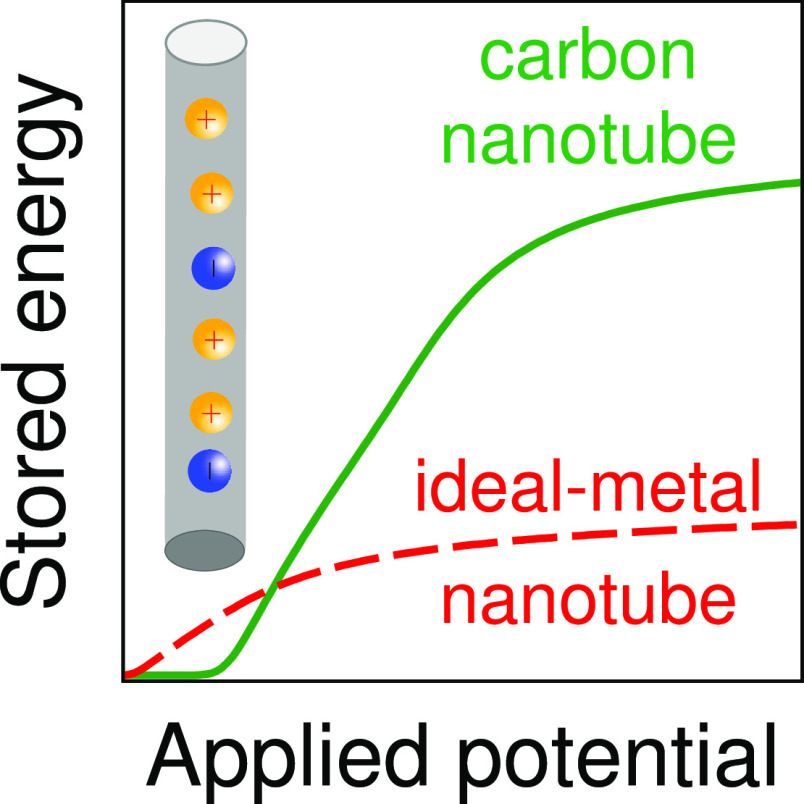

We present a theoretical analysis of charge storage in electrochemical capacitors with electrodes based on carbon nanotubes. Using exact analytical solutions supported by Monte Carlo simulations, we show how the limitations of the electron density of states in such low-dimensional electrode materials may help boost the energy stored at increased voltages. While these counterintuitive predictions await experimental verification, they suggest exciting opportunities for enhancing energy storage by rational engineering of the electronic properties of low-dimensional electrodes.

Since the pioneering work by Gerischer^1^ on electrical double-layer (EDL) capacitors with graphite electrodes, it has become clear that the ability to accommodate electrons subject to the available density of states (DOS) in an electrode influences its electrochemical properties. The limitations of the electron DOS are particularly strong in low-dimensional electrode materials, for example, those based on graphene sheets or carbon nanotubes. Such limitations lead to the so-called quantum capacitance, the quantity controlling the ability to accumulate charge on the electrode in response to the electrode’s potential,^2^ that can dramatically affect the total capacitance of an electrode–electrolyte system. With the boom in the use of various forms of low-dimensional nanostructured electrode materials,^3,4^ it is evident that each type of such an electrode may have its own electron DOS, and hence, the quantum capacitance should be considered separately for each case study. Nevertheless, in many simulation and theoretical studies, supercapacitor electrodes have still been modeled as perfect metals characterized by infinite quantum capacitance.

Studies of the effects of finite quantum capacitance have been mainly limited to flat electrodes^5−8^ and electrolytes outside of carbon nanotubes (CNTs).^9−12^ The main conclusion was that the quantum capacitance decreases the total capacitance. This conclusion supposedly follows from the capacitance in series expression *C*^–1^ = *C*_q_^–1^ + *C*_IL_^–1^ applied to an electrode–electrolyte system, where *C* is the total capacitance and *C*_q_ and *C*_IL_ are the quantum and EDL capacitances, respectively. It suggests that a finite *C*_q_ decreases the total capacitance *C* and hence energy storage, motivating researchers to seek methods for enhancing the quantum capacitance.^13−15^

Herein, we show for singe-wall carbon nanotubes (CNTs) that a nontrivial distribution of the electrostatic potential between the nanotube and electrolyte subsystems may lead to counterintuitive effects such as an enhancement of the capacitance and energy storage at increased voltages due to low quantum capacitance. Moreover, we demonstrate that, perhaps surprisingly, semiconducting CNTs can also increase the stored energy density at reasonably high (experimentally achievable) applied voltages.

Modeling real CNT-based electrodes^16−18^ is computationally challenging and requires the solution of a nontrivial quantum mechanical problem to find the distribution of electrons and electrostatic potential in the system. In this work, we consider a more straightforward problem when a potential *u* is applied at the outer surfaces of the nanotubes (Figure 1a). We assume that *u* is constant along the nanotube and use the analytical expression by Mintmire and White^19^ for the CNT’s DOS to compute the electric charge and quantum capacitance *C*_q_ as functions of gate voltage *u*_q_ (section S1 of the Supporting Information). The Mintmire–White formula provides an appropriate description for the DOS in most cases and compares favorably with quantum density functional theory (DFT) and other calculations.^19,20^Figure 1b shows two typical examples. For a metallic or quasi-metallic CNT, the capacitance *C*_q_ (per surface area) is constant in the first subband, exhibits a peak at its boundary due to the van Hove singularity of the DOS,^19^ attains a nearly constant value in the second subband, etc. For a semiconducting CNT, the behavior is similar; however, the capacitance is zero inside the band gap, and the locations and widths of the subbands are different.

**Figure 1:**
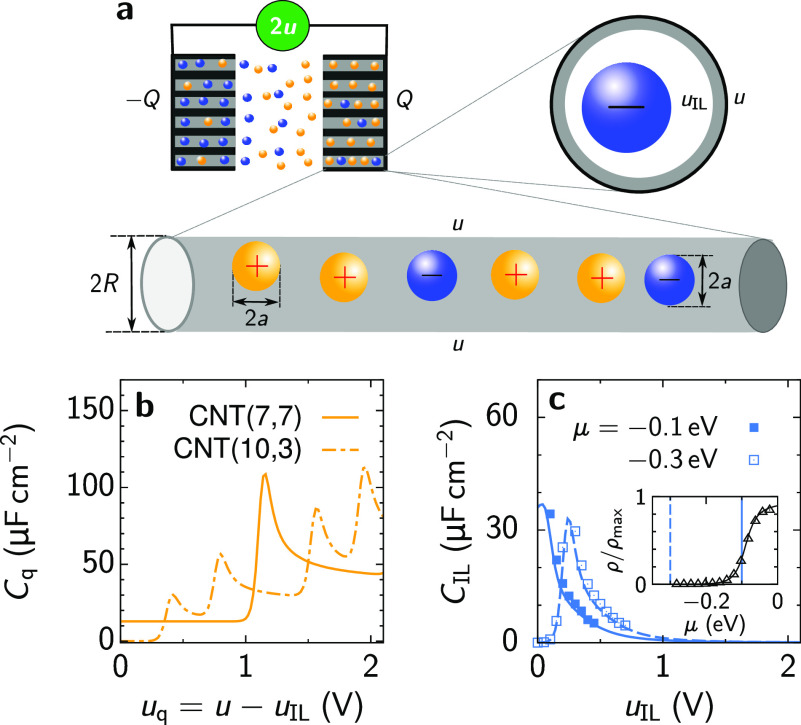
Ionic liquids in carbon nanotubes (CNTs) and examples of quantum and electrical double-layer capacitances. (a) Schematic of a supercapacitor with two nanoporous electrodes and ions of the same radius *a*. A potential *u* is applied to the outer surfaces of CNTs, as measured with respect to the bulk electrolyte; *u*_IL_ is the potential at the inner CNT surface. The bottom and top right cartoons show the side and top views, respectively, of a single CNT filled with ions. The radius of a CNT is *R* as measured to the center of the carbon atoms. (b) Examples of voltage-dependent quantum capacitance *C*_q_ calculated using the Mintmire–White formula for the CNT density of states^19^ (section S1). CNT(7,7) is metallic, and CNT(10,3) is semiconducting; the latter has zero capacitance inside the band gap (around zero voltage). The CNT radii are ≈0.47 and 0.46 nm, respectively. *T* = 300 K. (c) Examples of voltage-dependent electrical double-layer capacitance *C*_IL_ calculated using the analytical solution of ref (26) (lines) and 3D Monte Carlo simulations (symbols). The ion radius *a* = 0.25 nm, the tube radius *R* = 0.47 nm, the in-pore dielectric constant ε = 5, and *T* = 300 K. The inset shows the 1D total ion density at zero voltage as a function of the ion chemical potential μ; ρ_max_ = (2*a*)^−1^ is the maximum 1D density. The thin vertical lines show the μ values of −0.1 and −0.3 eV used in the main plot; these values correspond to a moderately ionophilic and ionophobic pore, characterized by high and vanishing in-pore ion density, respectively, as the inset demonstrates (see also Figure S1).

To compute the accumulated ionic charge and EDL capacitance *C*_IL_, we consider narrow CNTs accommodating only single rows of ions. Charging such single-file nanotubes can be described by one-dimensional (1D) analytical models.^21−26^ Most of these models assume that only nearest ions interact with each other, allowing the development of reliable, analytically tractable solutions for the charge density and capacitance. The nearest-neighbor assumption is reasonable given the confined ions are in the superionic state,^27^ meaning that electrostatic interactions are exponentially screened by the charge carriers of the confining walls.^27−31^ A recently developed 1D continuum model has an exact solution, which agrees quantitatively well with three-dimensional (3D) Monte Carlo (MC) simulations in a wide range of parameters^26^ (section S2). In Figure 1c, we present two typical examples of *C*_IL_ for an ionophilic and ionophobic pore, obtained by the exact solution (lines) and MC simulations (symbols), showing a decent agreement. The ionophilicity was controlled by the ion chemical potential (inset of Figure 1c and Figure S1), in which we included the ion’s electrostatic and dispersion interactions with the pore walls^26,32^ (section S2). The capacitance exhibits a maximum at (usually) non-zero voltage and decays quickly to zero with an increase in voltage as the nanotube becomes saturated with counterions.

To obtain the total capacitance *C*, we applied the charge electroneutrality condition to a CNT–electrolyte system
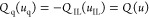
1where *Q*_q_ is the electric charge of the nanotube and *Q*_IL_ is the accumulated ionic charge, both per surface area, *u*_q_ = *u* – *u*_IL_ is the potential drop at the CNT wall, and *u*_IL_ is the potential at the inner CNT surface as seen by the confined ions (Figure 1a). Note that a positive *u*_q_ induces a positive charge on the CNT wall, i.e., *Q*_q_(*u*_q_ > 0) > 0, while a positive *u*_IL_ yields a negative ionic charge inside the nanotube, i.e., *Q*_IL_(*u*_IL_ > 0) < 0 (see section S1B). Having analytical expressions for *Q*_q_ and *Q*_IL_, we solved eq 1 numerically to find *u*_q_ and hence *Q*(*u*). The total capacitance of the CNT-electrolyte system (per surface area) is *C* = d*Q*/d*u*.

As an example, we consider a CNT with chiral indices *n*_1_ = *n*_2_ = 7 [i.e., (7,7) CNT], which is a metallic armchair nanotube with a radius of ≈0.47 nm. We chose the parameters such that the nonpolarized CNT is moderately filled with ions (Figure 1c). Figure 2a shows potentials *u*_q_ and *u*_IL_ = *u* – *u*_q_ as functions of voltage *u* applied to the CNT with respect to the bulk electrolyte. We find that *u*_q_ increases more strongly with *u* than *u*_IL_ does and eventually saturates at high voltages, while *u*_IL_ remains low and starts increasing only at *u* ≈ 1 V (Figure 2a). Because *C*_q_ ≪ *C*_IL_ at low voltages, the CNT wall requires a much higher voltage to achieve the same charge as the ionic system at a relatively low *u*_IL_. Of course, the resulting total capacitance is smaller than *C*_q_ and *C*_IL_ and, in fact, satisfies
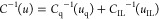
2Figure 2b shows *C*_q_ and *C*_IL_ evaluated at *u*_q_ and *u*_IL_, respectively, and the total capacitance *C*(*u*). Because *u*_IL_ < *u*, the EDL capacitance (*C*_IL_) stretches to larger applied potential differences (compare Figure 2b with Figure 1c or 2c), which has important consequences for energy storage, as we discuss below.

**Figure 2:**
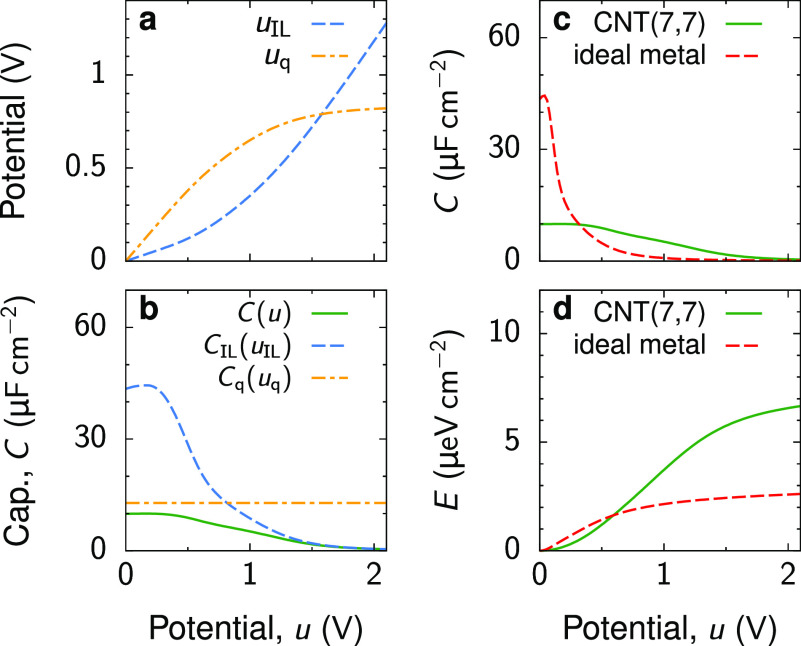
Charge storage in electrolyte-filled metallic CNTs. (a) Potentials *u*_IL_ and *u*_q_ = *u* – *u*_IL_ (see Figure 1a) as functions of voltage *u* applied to a (7,7) CNT with respect to the bulk electrolyte. (b) Quantum (*C*_q_) and electrical double-layer (*C*_IL_) capacitances evaluated at *u*_q_ and *u*_IL_, respectively, showing that the total capacitance *C* is smaller than *C*_q_ and *C*_IL_; *C* is determined by eq 2. (c) Comparison of the capacitance of a (7,7) CNT and of a nanotube of the same radius as a (7,7) CNT but with perfectly metallic walls. (d) Energy stored in a (7,7) CNT and in the metallic nanotube of the same radius. Although the CNT stores slightly less energy at low voltages, it provides a few times higher stored energy densities at moderate and high voltages. In all plots, the ion radius *a* = 0.25 nm, the tube radius *R* = 0.47 nm, the in-pore dielectric constant ε = 5, and *T* = 300 K. The ion chemical potential μ of −0.1 eV corresponds to a moderately ionophilic pore (inset of Figure 1c and Figure S1). For a comparison with ionophobic and strongly ionophilic pores, see Figure S3.

In Figure 2c, we compare the total capacitance of the (7,7) CNT and the same-sized nanotube with perfectly metallic walls; the latter assumes the infinite quantum capacitance and corresponds to the EDL capacitance alone. In line with previous work,^5−9,11,12^ the total capacitance is drastically reduced compared to the EDL capacitance due to the low quantum capacitance. However, this reduction occurs only at low voltages. At intermediate and high voltages, the capacitance of an ion-filled metallic nanotube practically vanishes because the nanotube becomes saturated with counterions. In contrast, the total capacitance of an ion-filled CNT is non-zero and remains relatively high up to ∼1.5 V.

We also calculated the energy stored in the nanotubes, which is given by
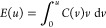
3Figure 2d shows that the metallic nanotube slightly outperforms the CNT at low voltages, but it falls far behind as the voltage increases. The stored energy nearly doubles at 1 V and triples at 2 V compared to that of the metallic nanotube. We observed similar behavior for other parameters (Figures S2 and S3), though the magnitude of the effect varied.

In addition to metallic CNTs, we also considered semiconducting CNTs. Such CNTs have zero quantum capacitance and charge carrier density inside the band gap, i.e., at voltages below the first van Hove singularity [*u*_vH_ ≈ 0.4 V for the (10,3) CNT of Figure 1c]. Note that the absence of charge carriers in the band gap implies that there is no image-charge effect, i.e., no screening of ion–ion interactions inside the nanotube in this voltage range. Increasing the gate voltage above *u*_vH_ shifts the Fermi energy of electrons and leads to a non-zero charge carrier density, giving rise to non-zero quantum capacitance and screened ionic interactions. For the sake of simplicity, we assumed the same screening for *u* > *u*_vH_ as for a metallic nanotube of the same radius. Although the actual screening might be slightly different, we do not expect any significant changes to our results. Bearing this in mind, we proceeded similarly as with metallic CNTs to calculate the total capacitance and stored energy density.

Unlike the charging of perfectly metallic nanotubes, a semiconducting CNT commences to charge at a non-zero voltage *u* ≈ *u*_vH_. Correspondingly, the accumulated charge, capacitance, and stored energy density vanish for *u* < *u*_vH_ (Figure 3). This behavior is reminiscent of ionophobic pores.^22,33,34^ Such pores are free of ions until a sufficiently high voltage is applied to overcome the ionophobicity barrier, which effectively shifts the charging to higher voltages, thereby enhancing energy storage according to eq 3.

**Figure 3:**
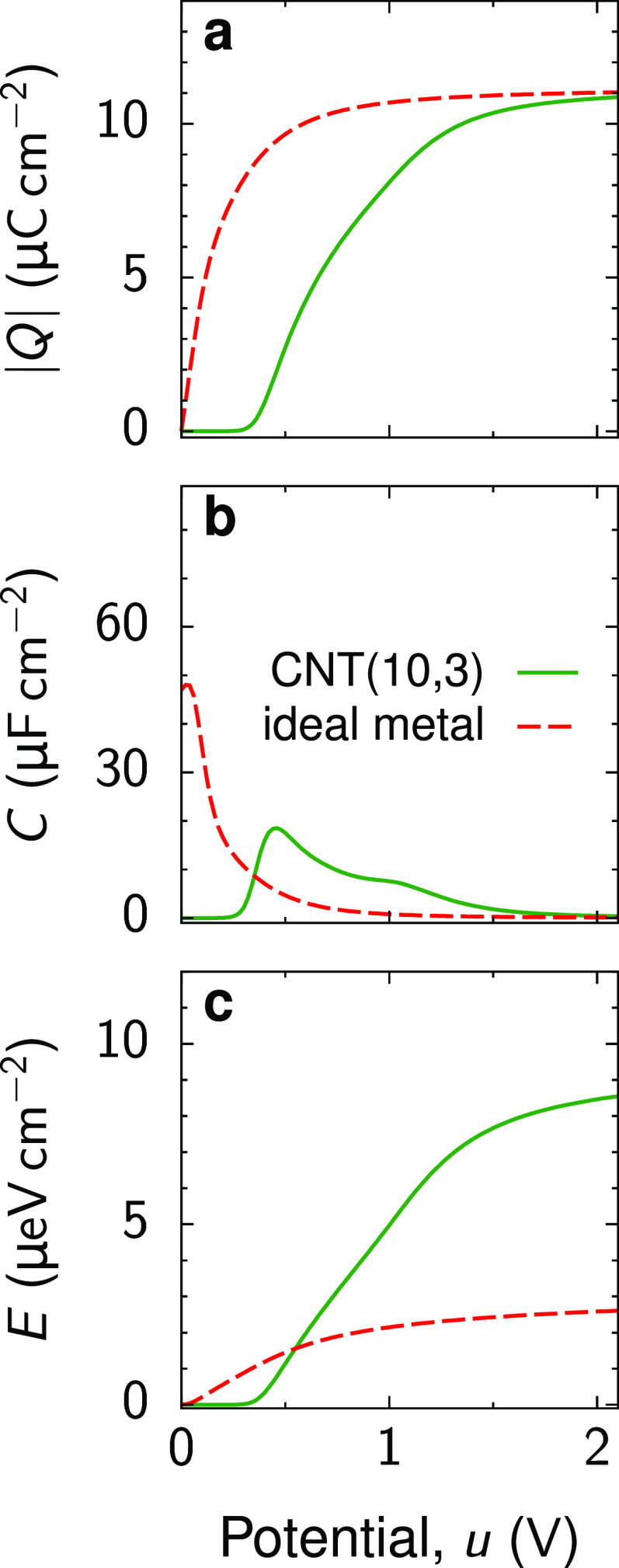
Charge storage in electrolyte-filled semiconducting CNTs. Comparison of (a) accumulated charge *Q*, (b) capacitance *C*, and (c) stored energy density *E* for a semiconducting (10,3) CNT and a nanotube of the same radius as the (10,3) CNT but with perfectly metallic walls. For the CNT, the accumulated charge, capacitance, and stored energy are zero for voltages *u* ≲ 0.4 V corresponding to the first van Hove singularity, which marks the band gap characterized by the vanishing quantum capacitance (Figure 1b). Although the charges stored in the (10,3) CNT and metallic nanotube are the same at high voltages, the former provides a few-fold higher stored energy densities. The ion radius *a* = 0.25 nm, the tube radius *R* = 0.46 nm, the in-pore dielectric constant ε = 5, *T* = 300 K, and the ion chemical potential μ = −0.1 eV. See also Figure S4.

A semiconducting nanopore is not necessarily empty. However, the vanishing quantum capacitance at low voltages shifts the charging process to voltages above *u*_vH_. Because of this shift, the energy stored in an ion-filled semiconducting CNT at moderate and high voltages is a few times higher than in the metallic nanotube of the same radius (Figure 3c), as more work is needed to charge it. This outcome is in line with the “pressing-the-spring” concept of ref (33).

To conclude, we have shown that, contrary to the traditional view, the low quantum capacitance of nanoporous electrodes can enhance energy storage by shifting the charging to higher voltages. We focused on narrow CNTs because of the available analytical solutions, allowing systematic and reliable analysis of the electrode charging under a wide range of applied voltages. However, similar principles can apply to other nanoporous electrodes. Thus, our results suggest, quite generally, “spoiling” a high-quantum capacitance electrode to enhance its energy storage at increased voltages, at which the EDL capacitance is low or vanishes. There are two main ingredients of this enhancement:

(1) The first is high EDL capacitance (*C*_IL_), which vanishes or decreases at moderate or high potential differences. Indeed, not only theory^33,35,36^ but also experimental work^37^ shows the reduced capacitance at high voltages (≳1 V) due to nanopore saturation.
(2) The second is small quantum capacitance (*C*_q_ ≪ *C*_IL_), or even vanishing *C*_q_ at low voltages. One example we have discussed is a CNT. Other low-dimensional materials, such as graphene-based electrodes, also have a small quantum capacitance.^38^ The quantum capacitance of carbon materials can be altered, e.g., via doping, but instead of enhancing,^13−15^ the goal is to reduce it.

Thus, we have demonstrated that exploiting the properties of quantum capacitance of low-dimensional electrodes can provide exciting opportunities to enhance capacitive energy storage even several times. With that said, the results of this work are still preliminary and await experimental verification. The predictions of the ideal theory can be affected by a pore size distribution of carbon electrodes,^39^ the carbonic nature of screening the ion–ion interactions inside pores,^29,31^ the pore shape and size,^25,40,41^ interpore ion–ion interactions,^42^ electron–electron correlations,^43^ and modification of the density of state of CNTs by in-pore ions^44,45^ and neighboring CNTs.^20^ However, we expect these factors to affect our main conclusions quantitatively but not qualitatively. We thus hope our results motivate further computational and experimental work with the ultimate goal of improving the energy storage ability of these ecologically friendly devices.

## Supporting Information Available

Derivations of the quantum and electrical double-layer capacitances, details of Monte Carlo simulations, and supplementary plots showing ion densities, capacitances, and energy densities for various types of pores (PDF)
Transparent Peer Review report available (PDF)
