# Correction to “Less is More: Can Low Quantum Capacitance Boost Capacitive Energy Storage?”

**DOI:** 10.1021/acs.jpclett.6c01828

**Published:** 2026-06-25

**Authors:** Taras Verkholyak, Andrij Kuzmak, Alexei A. Kornyshev, Svyatoslav Kondrat

There is a typographical error in the energy units on the *y*-axis of Figures 2d and 3c in the main text, as well as Figures S2d–f and S3d–f in the Supporting Information. The correct units are μJ cm^–2^.

This typographical error does not affect any results, interpretations, or conclusions presented in the paper.

