# Supplementary material for: Less Is More: Can Low Quantum Capacitance Boost Capacitive Energy Storage?
Source: J Phys Chem Lett. 2022 Nov 18;13(47):10976–80. doi: 10.1021/acs.jpclett.2c02968 (PMC9720744; doi:10.1021/acs.jpclett.2c02968)
Supplement: Supplementary file 1 — jz2c02968_si_001.pdf [file jz2c02968_si_001.pdf]

Supplementary Material to: "Less is More: Can Low Quantum Capacitance Boost Capacitive Energy Storage?"

Taras Verkholyak,[1] Andrij Kuzmak,[2] Alexei A. Kornyshev,[3,4] and Svyatoslav Kondrat[5,6]

[1]*Institute for Condensed Matter Physics, National Academy of Sciences of Ukraine, Svientsitskii Street 1, 79011 Lviv, Ukraine*

[2]*Department for Theoretical Physics, I. Franko National University of Lviv, Lviv, Ukraine*

[3]*Department of Chemistry, Molecular Sciences Research Hub, White City Campus, London W12 0BZ, United Kingdom*

[4]*Thomas Young Centre for Theory and Simulation of Materials, Imperial College London, South Kensington Campus, London SW7 2AZ, United Kingdom*

[5]*Institute of Physical Chemistry, Polish Academy of Sciences, 01-224 Warsaw, Poland*

[6]*Institute for Computational Physics, University of Stuttgart, Stuttgart, Germany*

CONTENTS

S1. Quantum capacitance of carbon nanotubes S2
- A. Density of states S2
- B. Charge and capacitance S3
- C. Numerical calculations S3

S2. Electrical double-layer capacitance of single-file pores S5
- A. Exact analytical solution of a continuum 1D model S5
- B. 3D Monte Carlo simulations S6

S3. Supplementary plots S8

References S12

S1. QUANTUM CAPACITANCE OF CARBON NANOTUBES

A general single-wall carbon nanotube is specified by a pair of the chiral indices (n_1, n_2) and the basis vectors $\mathbf{a}_1$, $\mathbf{a}_2$ of the honeycomb lattice [1]. The length of the basis vectors $a = |\mathbf{a}_1| = |\mathbf{a}_2| = \sqrt{3}d$, where $d \approx 0.142$ nm is the distance between two carbon ions in the bond. These data determines the circumference of a nanotube completely:

$$L = a\sqrt{n_1^2 + n_2^2 + n_1 n_2} = d\sqrt{3(n_1^2 + n_2^2 + n_1 n_2)}, \tag{S1}$$

and its diameter

$$D = \frac{L}{\pi} = \frac{d}{\pi}\sqrt{3(n_1^2 + n_2^2 + n_1 n_2)}. \tag{S2}$$

We can also calculate the area per one carbon atom in a single layer sheet as $A = \frac{3\sqrt{3}}{4}d^2 = 2.62 \times 10^{-16}$ cm^2, and its inverse quantity, the density of carbon atoms $1/A = 3.8177 \times 10^{15}$ cm^{-2}.

A. Density of states

A carbon nanotube can show either the metallic behavior if $(n_1 - n_2)$ is an integer multiple of 3, or the semiconducting behavior otherwise. In Ref. [2] the density of states per carbon atom was found in a universal form:

$$\rho(E) = \frac{1}{\Lambda |V_{pp\pi}|} U\left(\frac{\Lambda E}{|V_{pp\pi}|}\right), \tag{S3}$$

where $\Lambda = \frac{D}{d} = \sqrt{3(n_1^2 + n_2^2 + n_1 n_2)}/\pi$ is the dimensionless ratio of the CNT diameter to the carbon-carbon (C-C) bond distance. We note that there is a typo in [2], where the wrong factor 2 is retained in the denominator. $V_{pp\pi}$ is the hopping amplitude of π electrons in the graphene sheet [3], which we took $|V_{pp\pi}| \approx 2.5$ eV according to Ref. [2]. In Eq. (S3), the following function is introduced:

$$U(E) = \frac{2\sqrt{3}}{\pi^2} \sum_{m=-\infty}^{\infty} g(E, \varepsilon_m), \tag{S4}$$

$$g(E, \varepsilon_m) = \begin{cases} |E|/\sqrt{E^2 - \varepsilon_m^2}, & |E| > |\varepsilon_m|, \\ 0, & |E| < |\varepsilon_m|, \end{cases} \tag{S5}$$

$$g(E, 0) = 1, \tag{S6}$$

$\varepsilon_m^2 = (3m+1)^2$ for semiconducting CNT ($(n_1 - n_2)$ is not an integer multiple of 3), $\varepsilon_m^2 = (3m)^2$ for metallic CNT ($(n_1 - n_2)/3$ is an integer).

B. Charge and capacitance

Following the standard procedure (see e.g. Sec. 6.4 of [4]) the charge density per one carbon atom can be written as follows

$$Q(u) = e\int_{-\infty}^{0} dE\tilde{\rho}(E)[1 - f(\beta(E-\mu_e))] - e\int_{0}^{\infty} dE\tilde{\rho}(E)f(\beta(E-\mu_e)), \tag{S7}$$

where the first and second terms refer to the contributions of holes and electrons, and $\mu_e = -eu$ is the electrochemical potential of the conduction electrons. Here we use the notation u for the voltage, e for the proton charge, and $f(\beta E) = 1/(\exp(\beta E)+1)$ for the Fermi-Dirac function. In addition we introduce $\tilde{\rho}(E) = \frac{1}{e}\rho(E/e)$ to make the calculation in SI units.

After some algebra, we get for the surface charge density

$$\begin{aligned} Q_q = \frac{Q}{A} &= \frac{e}{A}\int_0^{\infty} dE\tilde{\rho}(E)[f(\beta(E-eu)) - f(\beta(E+eu))], \\ &= \frac{2\sqrt{3}}{\pi^2\Lambda|V_{pp\pi}|A}\sum_{m=0,\pm1,\ldots}\int_0^{\infty} dE g\left(\frac{\Lambda E}{e|V_{pp\pi}|}, \varepsilon_m\right)[f(\beta(E-eu)) - f(\beta(E+eu))], \end{aligned} \tag{S8}$$

and find the quantum capacitance as

$$\begin{aligned} C_q = \frac{dq}{du} &= -\frac{e^2}{A}\int_0^{\infty} dE\tilde{\rho}(E)[f'(\beta(E-eu)) + f'(\beta(E+eu))], \\ &= -\frac{2\sqrt{3}}{\pi^2\Lambda|V_{pp\pi}|}\frac{e\beta}{A}\sum_{m=0,\pm1,\ldots}\int_0^{\infty} dE g\left(\frac{\Lambda E}{e|V_{pp\pi}|}, \varepsilon_m\right)[f'(\beta(E-eu)) + f'(\beta(E+eu))], \end{aligned} \tag{S9}$$

where $f'(\beta E) = \frac{df(\beta E)}{d\beta E} = -\frac{1}{4\cosh^2\frac{\beta E}{2}} = -\frac{\exp(\beta E)}{(\exp(\beta E)+1)^2}$.

Note that $Q_q > 0$ for positive u, which follows from eq. (S8) because $f(\beta(E-eu)) > f(\beta(E+eu))$ in this case.

C. Numerical calculations

In case of numerical calculation it is useful to work with the dimensionless variables defined as follows: $E' = \frac{\Lambda}{e|V_{pp\pi}|}E$, $u' = \frac{\Lambda}{e|V_{pp\pi}|}u$, $T' = \frac{\Lambda}{e|V_{pp\pi}|}T$, $\beta' = \frac{e|V_{pp\pi}|}{\Lambda}\beta$. Then, the

equations above can be rewritten as

$$Q_q = \frac{2\sqrt{3}}{\pi^2\Lambda^2}\frac{e}{A}\sum_{m=0,\pm1,\ldots}\int_0^\infty dE' g\left(E',\varepsilon_m\right)\left[f(\beta'(E'-eu')) - f(\beta'(E'+eu'))\right],$$

$$C_q = -\frac{2\sqrt{3}}{\pi^2\Lambda^2}\frac{e^2\beta}{A}\sum_{m=0,\pm1,\ldots}\int_0^\infty dE' g\left(E',\varepsilon_m\right)\left[f'(\beta'(E'-eu')) + f'(\beta'(E'+eu'))\right]. \quad \text{(S10)}$$

Let us calculate the quantum capacitance of a metallic nanotube ($(n_1 - n_2)/3$ is integer) at zero voltage. In this case, we can restrict the calculation by the lowest conduction band $m = 0$. It is straighforward to show that

$$\int_0^\infty dE' f'(\beta' E') = \frac{1}{2\beta'}$$

Introducing this expression to Eq. (S10), we have

$$C_q(u=0) = \frac{2\sqrt{3}}{\pi^2\Lambda^2}\frac{e^2\beta}{A}\frac{1}{\beta'} = \frac{2\sqrt{3}e}{\pi^2\Lambda|V_{pp\pi}|A}. \quad \text{(S11)}$$

The capacitance per unit length can be also calculated in the nanotube:

$$C_q^{1D}(u=0) = C_q(u=0)\pi D = C_q(u=0)\pi\Lambda d = \frac{2\sqrt{3}ed}{\pi|V_{pp\pi}|A}. \quad \text{(S12)}$$

It is clear that within this approach, the linear capacitance does not depend on the diameter of the metallic nanotube, although the ab-initio simulations show some dependence on the chirality indices of CNTs [5]. For instance, eq. (S12) gives $C_q^{1D}(u=0) = 0.378\,\mathrm{fF\,\mu m^{-1}}$. Parkash and Goel[5] obtained with ab-initio calculations (see Table 2 in ref. [5]): $C_q^{1D}(u = 0) = 0.366\,\mathrm{fF\,\mu m^{-1}}$ for the $(12, 0)$ tube, $C_q^{1D}(u = 0) = 0.388\,\mathrm{fF\,\mu m^{-1}}$ for the $(9, 0)$ tube, and $C_q^{1D}(u = 0) = 0.207\,\mathrm{fF\,\mu m^{-1}}$ for the $(9, 9)$ tube.

S2. ELECTRICAL DOUBLE-LAYER CAPACITANCE OF SINGLE-FILE PORES

A. Exact analytical solution of a continuum 1D model

We consider a cylindrical nanopore of radius R (measured to the centers of the wall atoms), with a potential difference u applied between the inner pore surface and a bulk electrolyte (fig. 1a in the main text). We assume the nanopore is so narrow that it can only accommodate a single row of ions, which we model as equally-sized monovalent charged hard spheres of radius a. This single-file pore model can be mapped onto an exactly-solvable one-dimensional model of interacting particles with electrochemical potentials $\mu_\pm = \mu \mp eu$, assuming the same chemical potential for cations (+) and anions (−); here e is the proton charge and u is the applied voltage.

An electrostatic interaction energy between ions of types α and γ located at $\boldsymbol{r}_1 = (0, r_1, 0)$ and $\boldsymbol{r}_2 = (z, r_2, \varphi)$ (in cylindrical coordinates) inside a metallic cylinder of radius R_e is [6]

$$\begin{aligned}\beta\psi_{\alpha\gamma}(\boldsymbol{r}_1, \boldsymbol{r}_2) &= \beta\psi_{\alpha\gamma}(z, r_1, r_2, \phi)\\ &= \frac{2\lambda_B}{R_\mathrm{e}} \sum_{m=0}^{\infty} A_n \cos(m\phi) \sum_{n=1}^{\infty} \frac{J_m(k_{nm} r_1/R_\mathrm{e}) J_m(k_{nm} r_2/R_\mathrm{e})}{k_{nm}[Y_{m+1}(k_{nm})]^2} e^{-k_{nm} z/R_\mathrm{e}},\end{aligned} \tag{S13a}$$

where $A_0 = 1$ and $A_m = 2$ for $m \neq 0$, J_m and Y_m are the Bessel functions of the first and second kind and k_{nm} is the nth positive root of J_m. The Bjerrum length $\lambda_B = \beta e^2/\varepsilon$ (in Gaussian units), where ε is the dielectric constant inside the pore and $\beta = (k_B T)^{-1}$ the inverse temperature (k_B is the Boltzmann constant and T is the temperature). In eq. (S13a), $R_\mathrm{e} = R - \delta$ is the location of the image surface screening the interactions. We took $\delta = 0.8$ Å [7, 8], which is slightly less than half of the radius of the wall (carbon) atom, $a_\mathrm{c} = 1.685$ Å.

For two ions on the symmetry axis of the nanotube, $r_1 = r_2 = 0$, eq. (S13a) simplifies to

$$\beta\psi_{\alpha\gamma}(z) = \frac{2\lambda_B}{R_\mathrm{e}} \sum_{n=1}^{\infty} \frac{\mathrm{e}^{-k_{n0} z/R_\mathrm{e}}}{k_{n0}[J_1(k_{n0})]^2}. \tag{S13b}$$

For large ion-ion separations, $z \gg R_\mathrm{e}$, eq. (S13b) is approximated remarkably well in a wide range of parameters by [9]

$$\beta\psi_{\alpha\gamma}(z) \approx \frac{3.08\lambda_B}{R_\mathrm{e}} \mathrm{e}^{-2.4z/R_\mathrm{e}}. \tag{S13c}$$

Because of the exponential screening of inter-ionic interactions (eq. (S13c)), interactions beyond nearest neighbours can be neglected. Such one-dimensional system of particles

interacting solely with their nearest neighbours can be solved exactly using the method presented in ref. [10, 11]. The equation of state is given by the following relation [11]:

$$\mathrm{e}^{2\beta\mu}(\eta_{++}^2 - \eta_{+-}^2) - 2\mathrm{e}^{\beta\mu}\cosh(\beta eu)\eta_{++} + 1 = 0, \tag{S14}$$

where

$$\eta_{\alpha\gamma}(s) = \int_0^\infty dz\, \mathrm{e}^{-sz-\beta\psi_{\alpha\gamma}(z)}, \tag{S15}$$

and $\psi_{\alpha\gamma}$ is given by eq. (S13b), $s = \beta p$, p is the pressure along the pore. Note that $\eta_{+-} = \eta_{-+}$ and that $\eta_{++} = \eta_{--}$ for the symmetric system considered here.

The ions densities are determined as follows:

$$\rho_\pm(s) = -\frac{1}{2}\frac{\mathrm{e}^{\beta\mu}(\eta_{++}^2 - \eta_{+-}^2) - \mathrm{e}^{\mp\beta eu}\eta_{++}}{\mathrm{e}^{\beta\mu}(\eta_{++}\eta'_{++} - \eta_{+-}\eta'_{+-}) - \cosh(\beta eu)\eta'_{++}}, \tag{S16}$$

where $\eta'_{\alpha\gamma} = \partial\eta_{\alpha\gamma}/\partial s$. The charge per surface area accumulated in a pore is $Q_{\mathrm{IL}} = e[\rho_+ - \rho_-]/(2\pi R)$ and the differential capacitance is $C_{\mathrm{IL}}(u) = -dQ_{\mathrm{IL}}/du$. The analysis of eq. (S16) shows that $\rho_+ < \rho_-$ for $u > 0$, implying a negative charge $Q_{\mathrm{IL}} < 0$ in this case.

B. 3D Monte Carlo simulations

We have performed grand canonical Monte Carlo (MC) simulations of a three dimensional model to validate the exact analytical solution. The chemical potentials of the 1D and 3D models, μ and $\mu_\pm^{3D}$, are related by [11]

$$\mu_\pm = \mu_\pm^{3D} - \bar{\psi}_\pm, \tag{S17}$$

where

$$\beta\bar{\psi}_\pm = -\ln\left(2\pi\int_0^{R-a_{\mathrm{c}}-a} \mathrm{e}^{-\beta\psi_\pm(r)} r\, dr\right). \tag{S18}$$

In eq. (S18), r is the radial coordinate, a_{c} is the radius of pore wall atoms, and $\psi_\pm$ is the interaction energy of a $\pm$ ion with the pore wall. We assume that $\psi_\pm$ is the same for cations and anions and consists of image-force interactions [6]

$$\beta\psi_\pm(r) = \frac{\lambda_B}{2\pi R_{\mathrm{e}}}\sum_{m=0}^{\infty} a_m \int_0^{2\pi} d\phi\, \cos(m\phi) \int_0^\infty d\xi\, \frac{I_m(\xi r/R_{\mathrm{e}})}{I_m(\xi)} K_0\left(\frac{\xi}{r}\sqrt{r^2 + R_{\mathrm{e}}^2 - 2rR_{\mathrm{e}}\cos\phi}\right), \tag{S19}$$

where $a_m = 1$ if $m = 0$ and $a_m = 2$ otherwise, $I_m(x)$ and $K_m(x)$ are the modified Bessel functions of the first and second kind, respectively. Note that our considerations can be straightforwardly extended to include dispersions or other interactions [11, 12].

In our simulations, we used potentials (S13a) to describe interactions between ions and potentials (S19) for the ions and the nanotube surface. All ions interacted sterically with each other and with the nanotube. Our simulations consisted of translational, Widom insertion/deletion [13] and molecular-type swap [14] moves. We performed 10^6 MC steps for equilibration and, depending on a system, from 10^6 to 10^7 steps in production runs. Periodic boundary conditions were applied in the direction along the nanotube. In all simulations, the tube length was 25 nm.

S3. SUPPLEMENTARY PLOTS

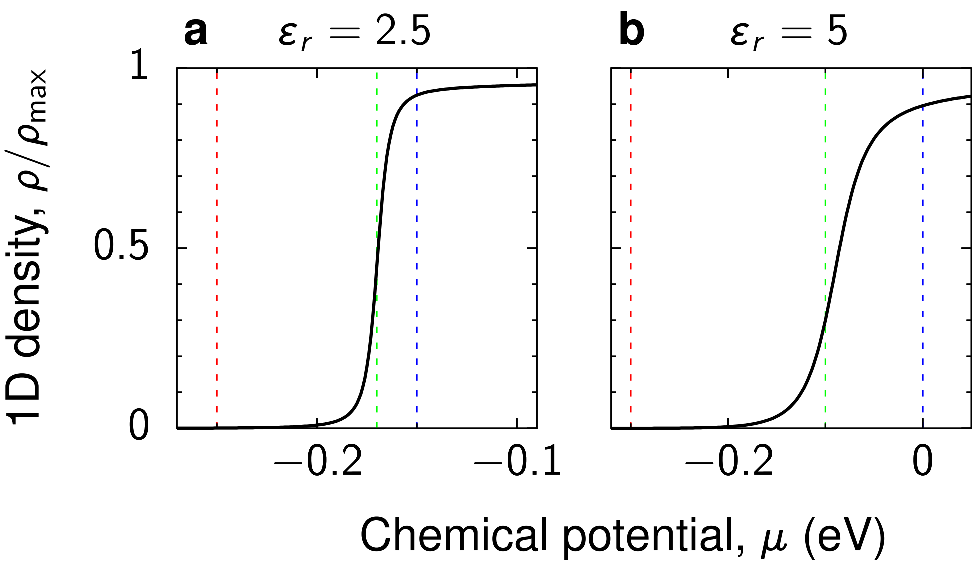

FIG. S1. **Non-polarised metallic CNT (7,7).** 1D ion density is shown as a function of the chemical potential μ. Ion radius $a = 0.25\,\text{nm}$, temperature $T = 300\,\text{K}$, and the in-pore dielectric constant $\varepsilon = 2.5$ (a) and $\varepsilon = 5$ (b). The thin vertical lines show the values of μ that we used to calculate the voltage dependence (see figs. S2 and S3 and fig. 2 in the main text).

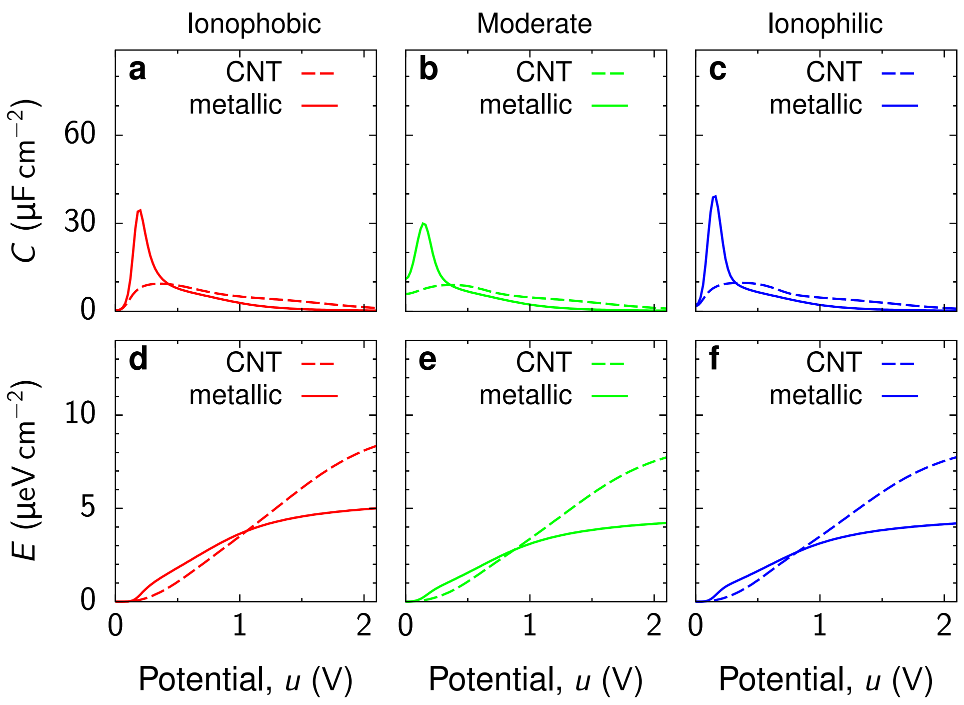

FIG. S2. **Charge storage in the (7,7) CNT for the in-pore dielectric constant $\varepsilon_r = 2.5$.** Capacitance and stored energy densities for (**a,d**) ionophobic (ion chemical potential $\mu = -0.25\,\mathrm{eV}$), (**b,e**) moderately ionophilic ($\mu = -0.17\,\mathrm{eV}$), and (**c,f**) strongly ionophilic ($\mu = -0.15\,\mathrm{eV}$) pores (see fig. S1). In all plots, the ions radius $a = 0.25\,\mathrm{nm}$, temperature $T = 300\,\mathrm{K}$, and the tube radius $R = 0.47\,\mathrm{nm}$ (the accessible radius $R_\mathrm{a} = R - a_\mathrm{c} = 0.3\,\mathrm{nm}$ and the screening surface radius $R_\mathrm{e} = 0.39\,\mathrm{nm}$, where $a_\mathrm{c} = 0.17\,\mathrm{nm}$ is the van der Waals radius of the carbon atom).

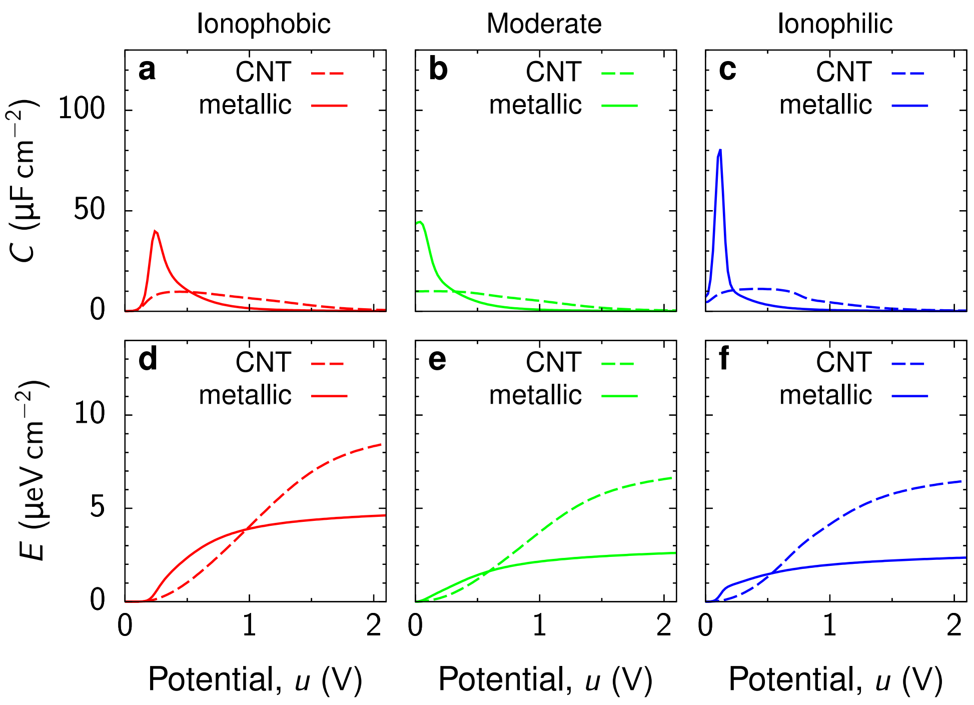

FIG. S3. **Charge storage in the (7,7) CNT for the in-pore dielectric constant $\varepsilon_r = 5$.** Capacitance and stored energy densities for (**a**,**d**) ionophobic (ion chemical potential $\mu = -0.3\,\mathrm{eV}$), (**b**,**e**) moderately ionophilic ($\mu = -0.1\,\mathrm{eV}$), and (**c**,**f**) strongly ionophilic ($\mu = 0\,\mathrm{eV}$) pores (see fig. S1). In all plots, the ions radius $a = 0.25\,\mathrm{nm}$, temperature $T = 300\,\mathrm{K}$, and the tube radius $R = 0.47\,\mathrm{nm}$ (the accessible radius $R_\mathrm{a} = R - a_\mathrm{c} \approx 0.3\,\mathrm{nm}$ and the screening surface radius $R_\mathrm{e} = 0.39\,\mathrm{nm}$, where $a_\mathrm{c} = 0.17\,\mathrm{nm}$ is the van der Waals radius of the carbon atom).

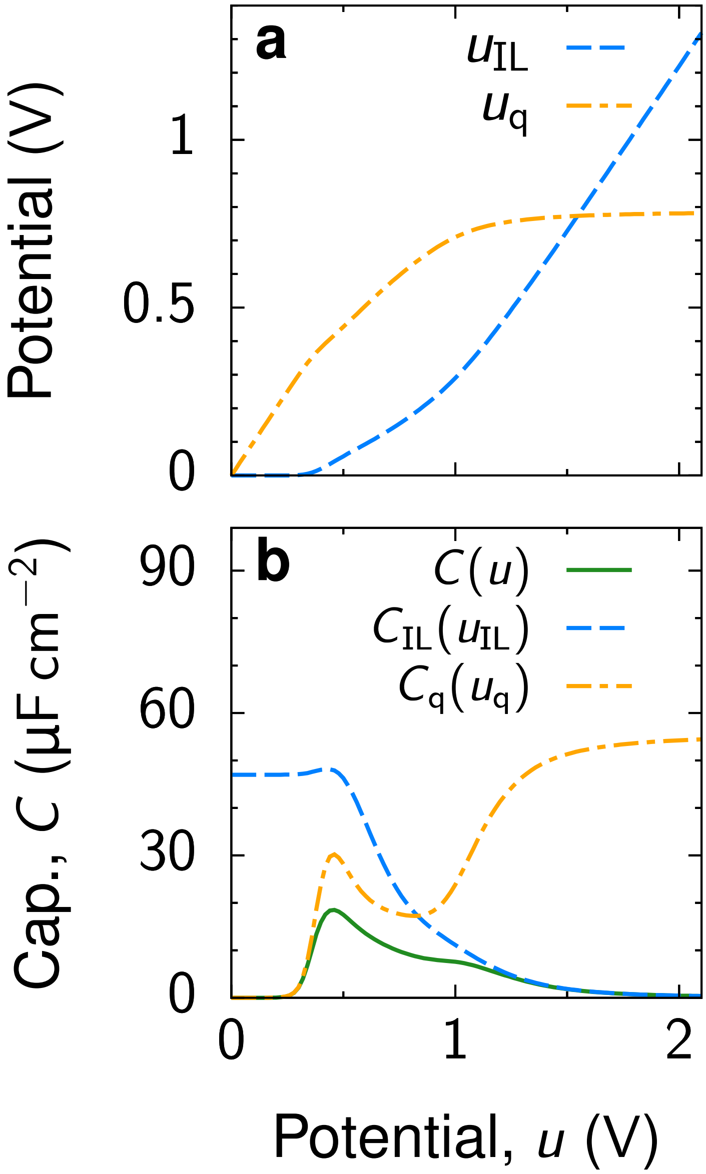

FIG. S4. **Charge storage in semi-conducting CNTs.** (**a**) Potentials $u_{\rm IL}$ and $u_{\rm q} = u - u_{\rm IL}$ (see fig. 1a in the main text) as functions of voltage u applied to a CNT (10,3) with respect to the bulk electrolyte. (**b**) Quantum ($C_{\rm q}$) and electrical double-layer ($C_{\rm IL}$) capacitances evaluated at $u_{\rm q}$ and $u_{\rm IL}$, respectively, showing that the total capacitance C is smaller than $C_{\rm q}$ and $C_{\rm IL}$; C is determined by $C^{-1}(u) = C_{\rm q}^{-1}(u_{\rm q}) + C_{\rm IL}^{-1}(u_{\rm IL})$. In all plots, the ions radius $a = 0.25\,{\rm nm}$, the tube radius $R = 0.46\,{\rm nm}$, the in-pore dielectric constant $\varepsilon = 5$, and temperature $T = 300\,{\rm K}$. The ion chemical potential $\mu = -0.1\,{\rm eV}$. See also fig. 3 in the main text.

REFERENCES

[1] M. Dresselhaus, G. Dresselhaus, and R. Saito, Physics of carbon nanotubes, Carbon **33**, 883 (1995), nanotubes.

[2] J. W. Mintmire and C. T. White, Universal density of states for carbon nanotubes, Phys. Rev. Lett. **81**, 2506 (1998).

[3] J. Mintmire, D. Robertson, and C. White, Properties of fullerene nanotubules, Journal of Physics and Chemistry of Solids **54**, 1835 (1993).

[4] H. Wong and D. Akinwande, *Carbon Nanotube and Graphene Device Physics*, Carbon Nanotube Graphene Device Physics (Cambridge University Press, 2011).

[5] V. Parkash and A. K. Goel, Quantum capacitance extraction for carbon nanotube interconnects, Nanoscale Res Lett **5**, 1424 – 1430 (2010).

[6] C. C. Rochester, A. A. Lee, G. Pruessner, and A. A. Kornyshev, Interionic interactions in electronically conducting confinement, ChemPhysChem **16**, 4121 (2013).

[7] C. Cagle, G. Feng, R. Qiao, J. Huang, B. G. Sumpter, and V. Meunier, Structure and charging kinetics of electrical double layers at large electrode voltages, Microfluid Nanofluid **8**, 703 (2009).

[8] N. D. Lang and W. Kohn, Theory of metal surfaces: Work function, Phys. Rev. B **3**, 1215 (1971).

[9] A. A. Kornyshev, The simplest model of charge storage in single file metallic nanopores, Faraday Discuss. **164**, 117 (2014).

[10] H. Longuet-Higgins, One-dimensional multicomponent mixtures, Mol. Phys. **1**, 83 (1958), https://doi.org/10.1080/00268975800100101.

[11] T. Verkholyak, A. Kuzmak, and S. Kondrat, Capacitive energy storage in single-file pores: Exactly solvable models and simulations, J. Chem. Phys. **155**, 174112 (2021).

[12] M. Janssen, T. Verkholyak, A. Kuzmak, and S. Kondrat, Optimising heat-to-electricity conversion with supercapacitors, J. Mol. Liq. To be published.

[13] B. Widom, Some topics in the theory of fluids, J. Chem. Phys. **39**, 2808 (1963).

[14] S. Kondrat, N. Georgi, M. V. Fedorov, and A. A. Kornyshev, A superionic state in nanoporous double-layer capacitors: insights from monte carlo simulations, Phys. Chem. Chem. Phys. **13**, 11359 (2011).
