# Supplementary material for: Less Is More: Can Low Quantum Capacitance Boost Capacitive Energy Storage?
Source: J Phys Chem Lett. 2022 Nov 18;13(47):10976–80. doi: 10.1021/acs.jpclett.2c02968 (PMC9720744; doi:10.1021/acs.jpclett.2c02968)
Supplement: Supplementary file 2 — jz2c02968_si_002.pdf [file jz2c02968_si_002.pdf]

jz-2022-029689.R1

Name: Peer Review Information for "Less is More: Can Low Quantum Capacitance Boost Capacitive Energy Storage?"

First Round of Reviewer Comments

Reviewer: 1

Comments to the Author

This is an interesting, enjoyable-to-read paper addressing the capacitance of single carbon nanotubes. It is suitable for publication in JPCL subject to the following feedback in response to the 3 questions you ask referees to address

1. What is the major advance reported in the paper?

The paper clarifies the link between capacitive effects and energy storage albeit using an extremely simplified model and making a very large number of assumptions, as the authors themselves recognise.

2. What is the immediate significance of this advance?

The paper will assist/stimulate the many experimental efforts in the area. It will have impact.

3. Technical suggestions. The paper needs to better clarify the meaning of 'quantum capacitance' at the outside to have better impact. Most essentially the paper needs a change of title to indicate the entirely speculative conclusions drawn re energy storage and that the work is entirely without experiment. Maybe 'Theoretical calculations hint that low quantum capacitance might boost ...."?

Otherwise tis is a good paper which meets very well the aims of JPCL

Reviewer: 2

Comments to the Author

The article is well written and consistently argued. The main argument is similar to

that proposed in ref. 34: The energy stored is higher, when a given charge is stored at a higher potential.

I have, however, a few questions which I would like to see discussed before publication:

The authors emphasize the important role of the image charge for the capacity.

Is this effect the same for conducting and for semiconducting tubes? Intuitively, I would expect the effect to be substantially larger on conducting tubes, so that C_IL would be smaller on semiconducting tubes, and the two contributions to the capacities would not be independent.

The application of a potential is supposed to induce a shift of the Fermi level, leaving the band edges unchanged. Is this correct? Do the inserted ions not lead to a deformation of the local electronic DOS?

Author's Response to Peer Review Comments:

Response to the Reviewers' reports

We thank all Referees for carefully reading our manuscript and for the comments and suggestions, which we have all taken into account and addressed in the manuscript. We refer to pages in the revised manuscript, where the changes have been made in response to the Reviewer's suggestions. We also provide a diff file, wherein all changes are highlighted.

RESPONSE TO REVIEWER #1

- *This is an interesting, enjoyable-to-read paper addressing the capacitance of single carbon nanotubes. It is suitable for publication in JPCL subject to the following feedback in response to the 3 questions you ask referees to address*

  We are grateful to the Referee for appreciating our work. We have amended the manuscript according to the Referee's suggestions and hope our manuscript is now suitable for publication in the Journal of Physical Chemistry Letters.

- *1. What is the major advance reported in the paper? The paper clarifies the link between capacitive effects and energy storage albeit using an extremely simplified model and making a very large number of assumptions, as the authors themselves recognise.*

  We agree with the Referee that our work clarifies the link between capacitive effects and energy storage. However, despite a large number of assumptions, we believe this effect is generic and can be observed in properly-designed experiments.

- *2. What is the immediate significance of this advance? The paper will assist/stimulate the many experimental efforts in the area. It will have impact.*

  Indeed, the formulated principles to enhance energy storage through the decrease in the quantum capacitance are quite general, although counterintuitive at a first glance, and we hope these results will motivate experimental and engineering studies.

- *3. Technical suggestions. The paper needs to better clarify the meaning of 'quantum capacitance' at the outside to have better impact. Most essentially the paper needs a change of title to indicate the entirely speculative conclusions drawn re energy storage*

and that the work is entirely without experiment. Maybe 'Theoretical calculations hint that low quantum capacitance might boost ....''?

We have followed the Referee suggestion and change the title of our manuscript to sound more speculative. We also defined the quantum capacitance in the introductory part of the manuscript (page 2).

RESPONSE TO REVIEWER #2

- *The article is well written and consistently argued. The main argument is similar to that proposed in ref. 34: The energy stored is higher, when a given charge is stored at a higher potential.*

  We thank the Referee for the positive assessment of our work.

- *The authors emphasize the important role of the image charge for the capacity. Is this effect the same for conducting and for semiconducting tubes? Intuitively, I would expect the effect to be substantially larger on conducting tubes, so that C_{IL} would be smaller on semiconducting tubes, and the two contributions to the capacities would not be independent.*

  We are sorry for being unclear. The Referee is correct that the image charge effect is substantial for conducting nanotubes, and less so at semiconducting ones, where it appears only after applying the potential above the first van Hove singularity. We have now made it clear in the manuscript (page 8).

- *The application of a potential is supposed to induce a shift of the Fermi level, leaving the band edges unchanged. Is this correct? Do the inserted ions not lead to a deformation of the local electronic DOS?*

  Indeed, an in-pore ion can modify the DOS of a CNT. However, in the absence of explicit quantum-mechanical calculations, we neglect this effect. To our knowledge, this assumption has been made in virtually all works on quantum capacitance in the context of EDL. We mention this effect explicitly in the concluding paragraph (page 9) and provide references that we found where the effect of 'impurities' on the DOS has been studied for graphene sheets.
